# Investigation of the selective color-changing mechanism of *Dynastes tityus* beetle (Coleoptera: Scarabaeidae)

**DOI:** 10.1038/s41598-020-80699-y

**Published:** 2021-01-12

**Authors:** Jiyu Sun, Wei Wu, Limei Tian, Wei Li, Fang Zhang, Yueming Wang

**Affiliations:** 1 Key Laboratory of Bionic Engineering (Ministry of Education, China), Jilin University, Changchun, 130022 People’s Republic of China; 2 University of Shanghai for Science and Technology, Shanghai, 200093 People’s Republic of China; 3 Research and Development Laboratory of Tescan China, Shanghai, 201112 People’s Republic of China; 4 Huzhou University, Huzhou, 313000 People’s Republic of China

**Keywords:** Biological techniques, Optics and photonics

## Abstract

Not only does the *Dynastes tityus* beetle display a reversible color change controlled by differences in humidity, but also, the elytron scale can change color from yellow-green to deep-brown in specified shapes. The results obtained by focused ion beam-scanning electron microscopy (FIB-SEM), show that the epicuticle (EPI) is a permeable layer, and the exocuticle (EXO) is a three-dimensional photonic crystal. To investigate the mechanism of the reversible color change, experiments were conducted to determine the water contact angle, surface chemical composition, and optical reflectance, and the reflective spectrum was simulated. The water on the surface began to permeate into the elytron via the surface elemental composition and channels in the EPI. A structural unit (SU) in the EXO allows local color changes in varied shapes. The reflectance of both yellow-green and deep-brown elytra increases as the incidence angle increases from 0° to 60°. The microstructure and changes in the refractive index are the main factors that influence the process of reversible color change. According to the simulation, the lower reflectance causing the color change to deep-brown results from water infiltration, which increases light absorption. Meanwhile, the waxy layer has no effect on the reflection of light. This study lays the foundation to manufacture engineered photonic materials that undergo controllable changes in iridescent color.

## Introduction

The varied colors of nature have a great visual impact on human beings. In nature, colors come from pigments, structural colors and bioluminescence, and the colors of beetle cuticles result from pigment colors, structural colors or a combination of both^1^. The principles of structural color and pigmentation proposed by scientists have revealed the mechanism of color formation. Pigment colors, also called chemical colors, are selectively absorbed by pigment molecules. The color is displayed by reflection and transmission of light at specific wavelengths. Structural colors are produced by the interaction of microstructures and light. And the mechanisms of structural color are thin film interference, diffraction grating, scattering and photonic crystal^2^. Beetles exhibit a great variety of structural colors, including green, blue, orange, white and others, which have been studied extensively^3–7^. The Japanese jewel beetle exhibits multilayer interference that allows it vary in color with different viewing angles^8^. *Chrysina gloriosa* exhibits circularly polarized reflection and shows different colors under left- and right-circular polarizers^3^. *Aglyptinus tumerus* contains a Bragg grating that diffracts visible white light into a rainbow^2^. The elytra of the weevil *Eupholus magnificus* are marked by yellow and blue bands with ordered 3D photonic crystal structures^9^.

Other than that, the interesting reversible color-change in beetles have introduced new insights into the mechanisms of structural color. The blue scales on the cuticle of the male beetle *Hoplia coerulea* can absorb water, with two-dimensional photonic crystal structure, which have been shown to be responsible for the beetle’s bright blue coloration, reversibly turn to emerald green with increasing water contents^4^. It is explained by reflectance change in the complete filling of the voids of the photonic structure with liquid water (refractive index 1.33). The elytra from *Dynastes hercules* appear khaki-green in a dry atmosphere and turn into black under high humidity levels also resulting from that the empty holes of three-dimensional photonic crystals are filled with water under high humidity^10^. *Charidotella egregia* is able to actively and reversibly toggle between the gold (resting state) and the diffuse-red (disturbed state) by the variation of light scattering in randomly distributed patches^11^. Scales of *Tmesisternus isabellae* reversibly change their coloration from golden in the dry state to red in the wet state, due to both the swelling of layers and the infiltration of waver into the air voids with water absorption and evaporation^12^. *Cryptoglossa verrucose* exhibits distinct color phases that reversibly varies from blueish-white to black owing to light refraction on the variation of surface network^13^. All of these complex and changeable examples of structural color are formed by reflection, scattering and refraction, which result from light–light and light–medium interactions in the microstructures.

For photonic nanoarchitectures, they are more complicated as one of fundamental optical formation with 2-D or 3-D microstructures. Photonic crystals are common in beetles and can achieve optical effects of reversible color change and angle dependence colors^11,13,14^. These color-generating structures are highly optimized by many millennia of evolution, which may influence the individual surviving as well as the chance to reproduce^15^. Different from periodic ordered nanostructure, 3-D photonic crystals with random network of interconnecting cuticular filaments were found existing in white beetle *Cyphochilus*, which appears to exhibit the efficient broadband optical scatter^1,16^. It is Amorphous photonic crystals that possess only short-range order structure owing to their interesting optical responses, such as isotropic photonic bandgaps, non-iridescent structural colors and light localization^17^. The extensive research efforts have been taken to investigate the photonic crystal structure (an artificial microstructure whose lattice size corresponds to the wavelength of light.) of biological origin of beetles for the potential application in engineering industry.

For the potential application in engineering, the establishment of microstructure models and theoretical calculations should be optimized. The theories of structural color have been well known. To obtain the structural characteristics, three-dimensional structures of elytra needs to be investigated. Focused ion beam-scanning electron microscopy (FIB-SEM) are mainly used to study biomedical materials and tissues, from small-scale cell to the larger scale interactions^18–20^. It can sequentially cut thin slices from a sample with a Ga ion beam and provide an SEM picture of the microstructure of the newly exposed section^21^. After the experiment, the three-dimensional structures are constructed by assembling serial SEM images of the sections. The three-dimensional structure of FIB data can represent the inner structure of a sample with higher resolution than the deduced structures obtained from two-dimensional pictures.

In this paper, FIB-SEM was used to investigate the microstructure of the *Dynastes tityus* elytron. The structural model was established from the high-resolution structural data of this system. Then, the mechanism of the interesting reversible color change of this beetle was explicated in the dry state and the wet state. These results will provide a reference for the design of controllable iridescent colored materials that respond to changes in the environment.

## Materials and methods

### Specimens

*Dynastes tityus* beetles (10 females) were transported to Shanghai, China from the eastern United States. The live beetles were fed with sugar jelly and kept in individual plastic compartments to prevent them from fighting with each other. All elytron samples were acquired from the live beetles and immediately prepared for experiments.

The specimen preparation steps for FIB-SEM were as follows. Six appropriate 2 mm × 1 mm × 1 mm elytron specimens were cut from anesthetized *D. tityus* adult beetles. Then, the specimens were immersed in 0.1 mol/L PB solution (phosphate buffer without sodium ion, pH = 7.4) containing 2.5% glutaraldehyde for 12 h. After anterior fixation, the specimens were washed with PB. A solution containing 3% potassium ferrocyanide in 0.3 M cacodylate buffer was mixed with an equal volume of 4% aqueous osmium tetroxide. The specimens were incubated in this solution for 1 h and then in fresh thiocarbohydrazide for 2 h. The target specimens were stained in uranyl acetate and lead citrate solution next. Finally, the specimens were washed with distilled water three times and dehydrated with ethanol six times for 20 min each.

### Surface morphology and interior microstructure

Elytron scales were dissected from female adult beetles to observe the surface morphology. Each specimen was fixed on a glass slide and viewed under stereoscopic microscope (SteREO Discovery V20, Zeiss, Germany) and confocal laser scanning microscope (CLSM; OLS3000, Olympus, Japan). The details of pits on elytron surface were obtained by environmental scanning electron microscope (ESEM; JEOL JSM-6700F, FEI Company, USA).

To determine the internal three-dimensional structure of the cuticle, FIB-SEM was performed using a FIB-SEM system (GAIA3, Tescan, Czech Republic). In addition, three-dimensional reconstruction was conducted by the three-dimensional visualization software Dragonfly Pro 2.0. The FIB-SEM system is equipped with a dual beam consisting of a scanning electron beam (SEM beam) and a focused ion beam (FIB). Each milling step of the ion beam was performed with 119 µA emission and 5 kV acceleration voltage. In case of possible structural damage to the cut edge by the scalpel, the middle position of the elytron surface was chosen for the tests. Serial sectioning was performed by 200 consecutive millings, each with a thickness of 30 nm. The depth of focus was 9.3 µm. The volume of each serial section was 12.7 µm × 6 µm × 9.3 µm.

### Contact angle measurements

The water contact angles of the elytron were measured by contact angle system (OCA20, Dataphysics, Germany) at 20 °C and 34% humidity. The static contact angle *θ* should be calculated on a highly flat surface. To ensure accurate results, the highest and flattest positions of the elytron were chosen for this experiment. In addition, all the specimens were fixed on the glass slides with modified acrylate adhesive to eliminate deviation caused by the arc of the elytra. The contact angle measurements were repeated 15 times with 5 samples collected from 5 live beetles. The volume of the water droplets was 2 µL. All the reported results of *θ* are the averages of the fifteen measurements. The changes in the water droplets on the surface were monitored at 2 min intervals for a total of 14 min.

### Surface chemical composition

Energy dispersive X-ray spectrometry (EDS) (EDS Inca X-Max, Oxford, England) was used to investigate the surface chemical composition of the elytron equipped with scanning electron microscope (EVO18, Zeiss, Germany). The analyses were performed at four different positions on fresh elytra. Before the measurements, the whole surface of each elytron was treated with ethyl alcohol to remove adhered sawdust.

### Optical measurements

Fresh elytron scales were a yellow-green color in the room environment at 28% humidity and 22 °C. These scales were fixed on glass slides prepared for optical measurements. After optical measurements were performed on yellow-green scales in low humidity, the scales were infiltrated by wet gauze until they became a deep-brown color. To observe the optical properties of deep-brown elytron, the deep-brown scales were moved to the test compartment immediately, and the testing was conducted as soon as possible before their color returned to yellow-green. The angular-dependent reflectance spectra were investigated with an angle-resolved spectroscopy system (ARM, IdeaOptics Instruments, China) and a 100 W halogen light source.

### Color simulation

In optical theory, the reflection and refraction of multilayer films occur at the junction of layers. And the reflection and refraction in each layer will be superimposed. The total reflection and refraction are the result of the superposition of countless reflections and refractions on the interface. The reflectivity coefficient and transmittance coefficient of the medium are obtained by obtaining the admittance of the medium based on Maxwell’s equations. Then, the reflectance of the model in the dry state and the wet state could be estimated by a matrix transformation. The reflectance of a thin-film multilayer can be calculated as follows^22^:

1 $$\left[ {\left. {\begin{array}{*{20}c} F \\ G \\ \end{array} } \right]} \right. = \left\{ {\left. {\prod\limits_{i = 1}^{K} {\left[ {\left. {\begin{array}{*{20}c} {\cos \delta_{i} } & {\frac{j}{{\eta_{i} }}\sin \delta_{i} } \\ {j\eta_{i} \sin \delta_{i} } & {\cos \delta_{i} } \\ \end{array} } \right]} \right.} } \right\}} \right.\left[ {\left. {\begin{array}{*{20}c} 1 \\ {\eta_{G} } \\ \end{array} } \right]} \right.,$$

2 $$R = rr^{ * } = \left( {\frac{{\eta_{0} F - G}}{{\eta_{0} F + G}}} \right)\left( {\frac{{\eta_{0} F - G}}{{\eta_{0} F + G}}} \right)^{ * } ,$$

where $\delta_{i} = 2\pi N_{i} d_{i} \cos \theta_{i} /\lambda$, $N_{i}$ is the optical admittance of the layer $i$ (The value is equal to the refractive index of the *i*th film.); $d_{i}$ is the thickness of the layer $i$; $\theta_{i}$ is the angle of incidence of the layer $i$; $\eta_{G}$ is the refractive index of the substrate; $\eta_{0}$ is the refractive index of air; and $R$ is the reflectance of a *Dynastes tityus* elytron.

### Ethics

This work complies with ethical guidelines at Jilin University.

## Results

### Color-changing and microstructure of elytra

**Figure 1:**
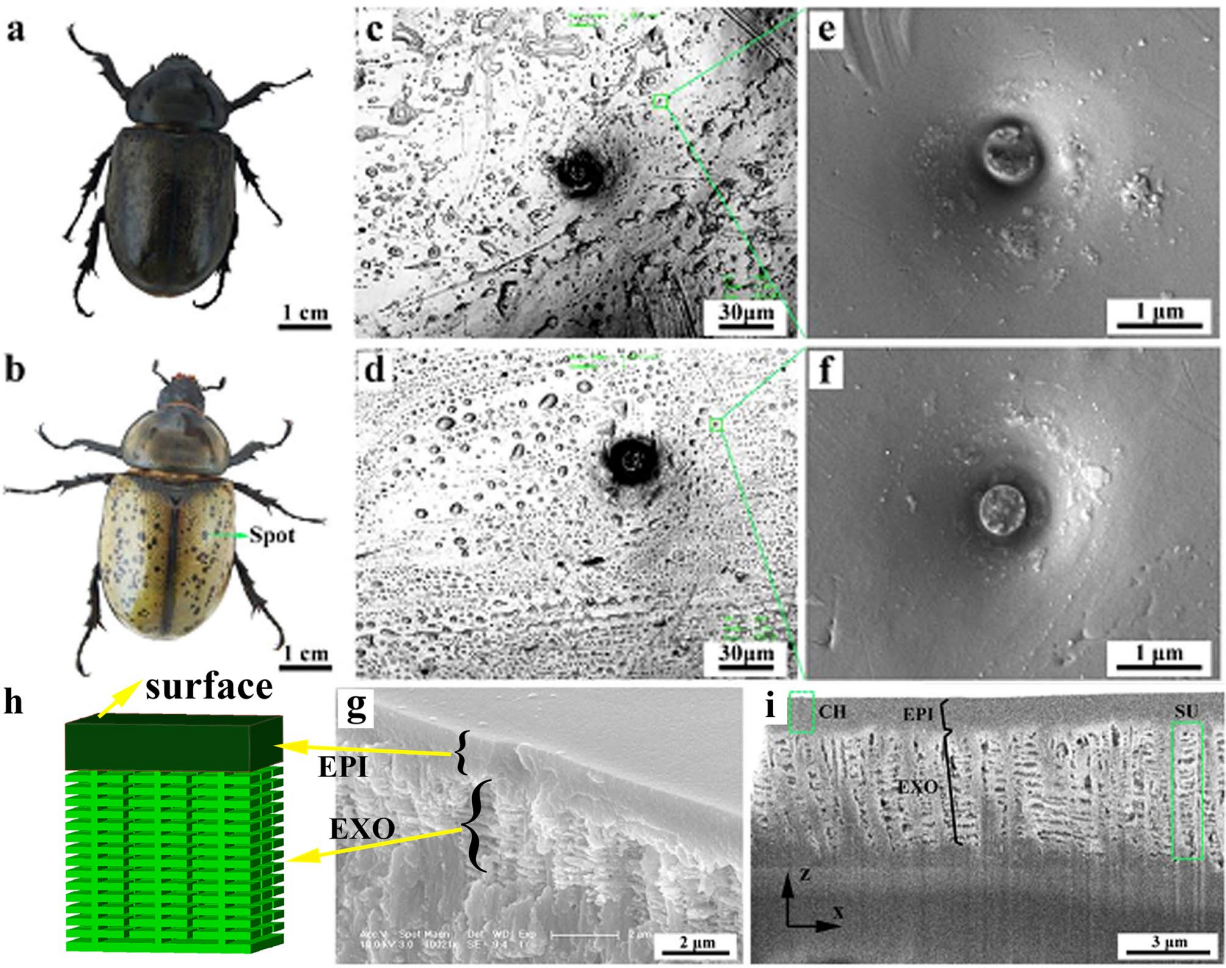
Two different color states of *D. tityus*: deep-brown in high humidity, 95% (**a**); yellow-green in low humidity, 22% (**b**). CLSM images of elytron surface morphology of spot (**c**) and yellow-green region (**d**). ESEM images of pits on spot (**e**) and yellow-green region (**f**). Cross-sectional ESEM images from liquid nitrogen fracture (**g**)^23^ and its 3D structure diagram (**h**). (**i**) FIB milling process. *EPI* epicuticle, *EXO* exocuticle, *CH* channel, *SU* structural unit.

*Dynastes tityus* can undergo a remarkable and reversible cuticular color change as the humidity varies. Figure 1 shows the visible difference between beetles in two environments with a great disparity in humidity. The elytron gradually turns deep-brown (Fig. 1a) in an environment with relatively high humidity of 95%, whereas it is yellow-green (Fig. 1b) in the normal room environment with a relative humidity of 22%. Figure 1c,d show the porous structure morphology on the surface of spot and yellow-green region of the elytron, with numerous randomly distributed pores of different sizes. Despite the existent pores, there are some pits on the surface of black spot and yellow-green region of elytron as shown in Fig. 1e,f, respectively. In the middle of pit, fractural like pillar was found. Figure 1g gives the photonic nanoarchitectures under epicuticle (EPI) of elytron interior^23^. Figure 1h shows a middle SEM image from the FIB milling process, which clearly reveals two different layers in the inner structure. The top layer is the EPI, which is approximately 1 μm in thickness and contains pore channels (CHs). The pits on spot and yellow-green region are entrance and exit of CH, which collect water and then absorb water molecules through fractural pillars to CH. These CHs cross the horizontal EPI in a vertical direction and act as waterpipes to transport water molecules from the outside world to inside the cuticle. Under the EPI, the structure of the exocuticle (EXO) in the elytron consists of longitudinal and transverse protein fibers. The ordered protein fibers display a netlike pattern. As shown in Fig. 1h, the structural unit (SU) can be clearly distinguished despite the destruction of some of its structural characteristics. The SU consists of a multilayer with nearly parallel individual layers.

**Figure 2:**
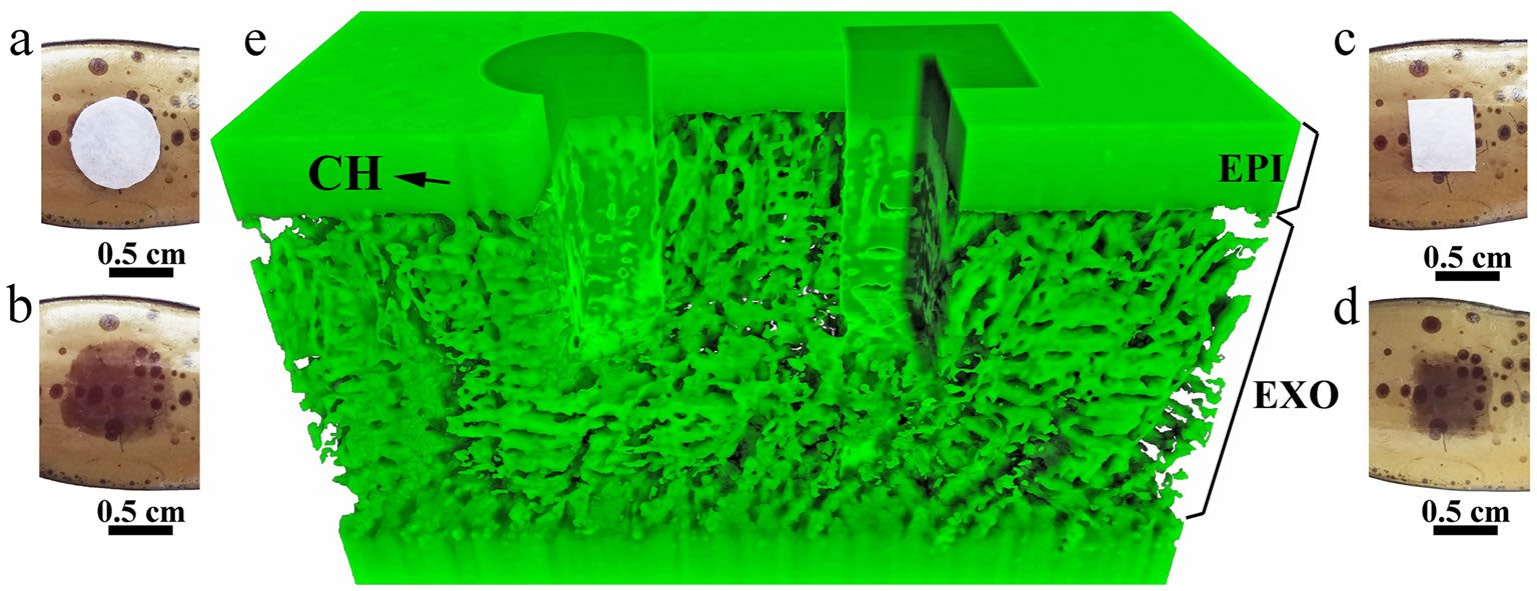
*Dynastes tityus* elytron infiltrated with water from circular (**a**) and square (**c**) wet papers. Local color changes occurred in corresponding circular (**b**) and square (**d**) shapes, which were included in the three-dimensional reconstruction of the microstructure in the FIB-SEM results (**e**) three-dimensional structure of microstructure additional details revealed by FIB.

To facilitate understanding the color change of the SU, circular and square shapes of local color change were designed, and a three-dimensional reconstruction of the microstructure of FIB-SEM results is shown in Fig. 2. Figure 2a,c shows the fresh elytron with circular and square papers soaked with water carefully placed on its surface. After 5–8 min, the elytron exhibited circular and square shapes of deep-brown color in the middle, while the rest of the elytron, interestingly, retained its yellow-green color, as shown in Fig. 2b,d. Specific parts of the elytron changed their color to form clear outlines corresponding to the different shapes of wet paper. The three-dimensional structure revealed by FIB provides additional details of the microstructure: the EPI is a permeable layer, and the EXO is a three-dimensional photonic crystal, as shown in Fig. 2e. EXO is densely arranged irregular multilayer structures without hollow structure. CH is the channel connecting the internal photonic crystal and outside. Photonic crystals consist of SU arranged periodically. They are closely connected and compact between different layers, making the elytron thick and hard. To reveal the difference in the internal microstructural state between deep-brown and yellow-green, this color change was simulated by reconstruction model.

### Variation of contact angles on the elytral surface

**Figure 3:**
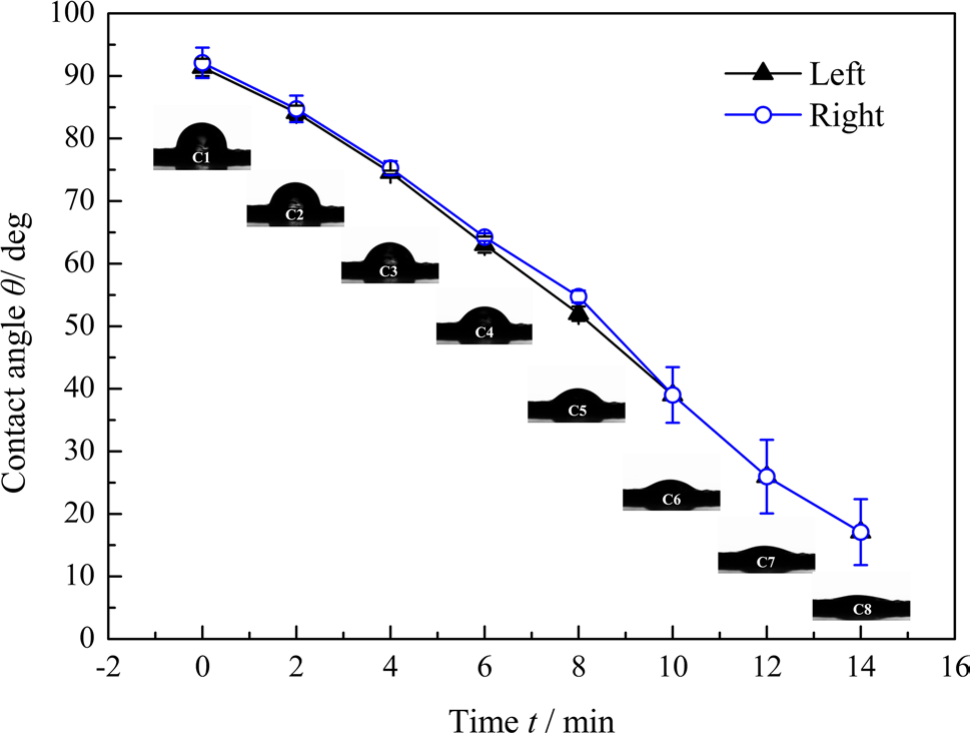
Relationship between contact angles and contact time on the elytron surface during contact angle measurements.

The elytra of beetles are known to be hydrophobic^24^. However, the elytra of *D. tityus* appear to be hydrophilic in light of their water-induced reversible color change. Water contact angle experiments were conducted to investigate the hydrophobicity or hydrophilicity of the elytral surface. The left and right contact angles measured for the elytra were 91.4 ± 1.36° and 92.1 ± 1.95°, respectively. During the contact time, the contact angles and the changes in the water drop were monitored 2 min a time as the drop settled on the surface of the elytron. Figure 3 shows pictures of the ongoing shape change and corresponding contact angle of the water droplet on the elytron surface. The beginning is the moment when the droplet first contacts the surface, and subsequent images were obtained every 2 min. The drop clearly became increasingly flat as time passed. The elytron resembles a sponge and can absorb the water on its surface. The left and right contact angles decreased from 91.4° to 17.1° and from 92.1° to 17.1°, respectively. The left and right angles displayed the same variation, indicating that the surface was flat enough for measurements. The contact angles were greater than 90° when the drop first fell to the elytron surface, showing that the surface was hydrophobic. After 2 min, the left and right contact angles were 84.1° and 84.7° respectively, implying that the hydrophobic surface had converted into a hydrophilic surface.

### Chemical composition of the elytral surface

The insect cuticle consists not only of chitin but also of other molecules such as protein, lipid and wax. As shown in Table 1, the elemental composition of the elytron surface of *D. tityus* was determined. The main elements are carbon (C), oxygen (O) and nitrogen (N) with atomic percentages of 48.69%, 32.55% and 16.77%, respectively. These elements originate from chitin, protein and wax produced by the copolymerization of *N*-acetyl-*β*-glucosamine and d-glucosamine^25^. The metallic elements copper (Cu), aluminum (Al), magnesium (Mg), calcium (Ca) and iron (Fe) are present in small amounts. Other non-metallic elements, sulfur (S), silicon (Si) and chlorine (Cl), mainly function in the formation of acids and salts with metallic elements.

**Table 1:** The chemical contents of the elytral surface of the beetle *D. tityus.*

Elements	C	N	O	Cu	Al	S	Mg	Ca	Fe	Si	Cl	K	Total
Atomic percentage	48.69	32.55	16.77	0.43	0.30	0.28	0.27	0.24	0.24	0.12	0.09	0.02	100
Weight percentage	42.06	32.08	19.30	1.97	0.59	0.65	0.47	0.69	0.97	0.24	0.22	0.05	

### Reflectance of color-changing elytron and color simulation

**Figure 4:**
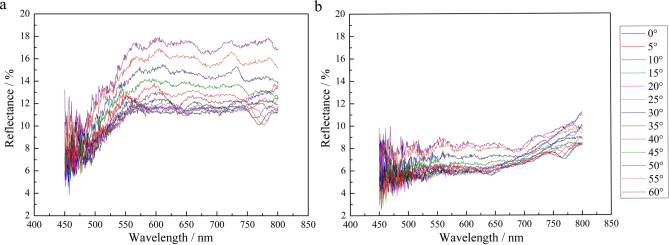
Measured light reflectance for *D. tityus* in the yellow-green dry state (**a**) and the deep-brown wet state (**b**) at incidence angles varying from 0° to 60° with an angular interval of 5°.

Figure 4 shows the reflectance of the elytron in dry atmosphere and when wet, obtained by optical measurements. The reflectance increased gradually as the incidence angle increased from 0° to 60°. No peak wavelength of the reflective light was observed for either yellow-green or deep-brown elytra. These results clearly show that the reflectance of yellow-green elytron scales in the dry state is higher than that of deep-brown scales in a wet atmosphere. As shown in Fig. 4a, the reflectance fluctuates in wavy curves at wavelengths between 550 and 800 nm. The fluctuation range is between 11 and 18%. For every incidence angle, the variation in reflective light follows a highly similar tendency. After the color change induced by high humidity, the reflective spectrum curves decrease to a value between a minimum of 5% and a maximum of 9%, as shown in Fig. 4b, which is much smaller than the variation range (11–18%) in a dry atmosphere. This is because that after water molecules enter the elytron, the voids of the photonic crystal structure inside the elytron are filled with water, resulting in a change in refractive index, from 1 in air to 1.33 in water. When the incident light enters the elytron, it will be refracted and reflected in the photonic crystal structure and water. Due to the high refracted index of water, less light will be reflected from the elytron. The reflective value increases with the increasing of measure angle, which is consistent with the change trend of dry atmosphere. Therefore, the photonic crystal structures still affect the reflection and refraction of incident light at different angles in elytron after filling with water. The elytron displays a reversible color change controlled by differences in humidity which is related with the water absorption expansion of the photonic crystal structure in the elytron.

**Figure 5:**
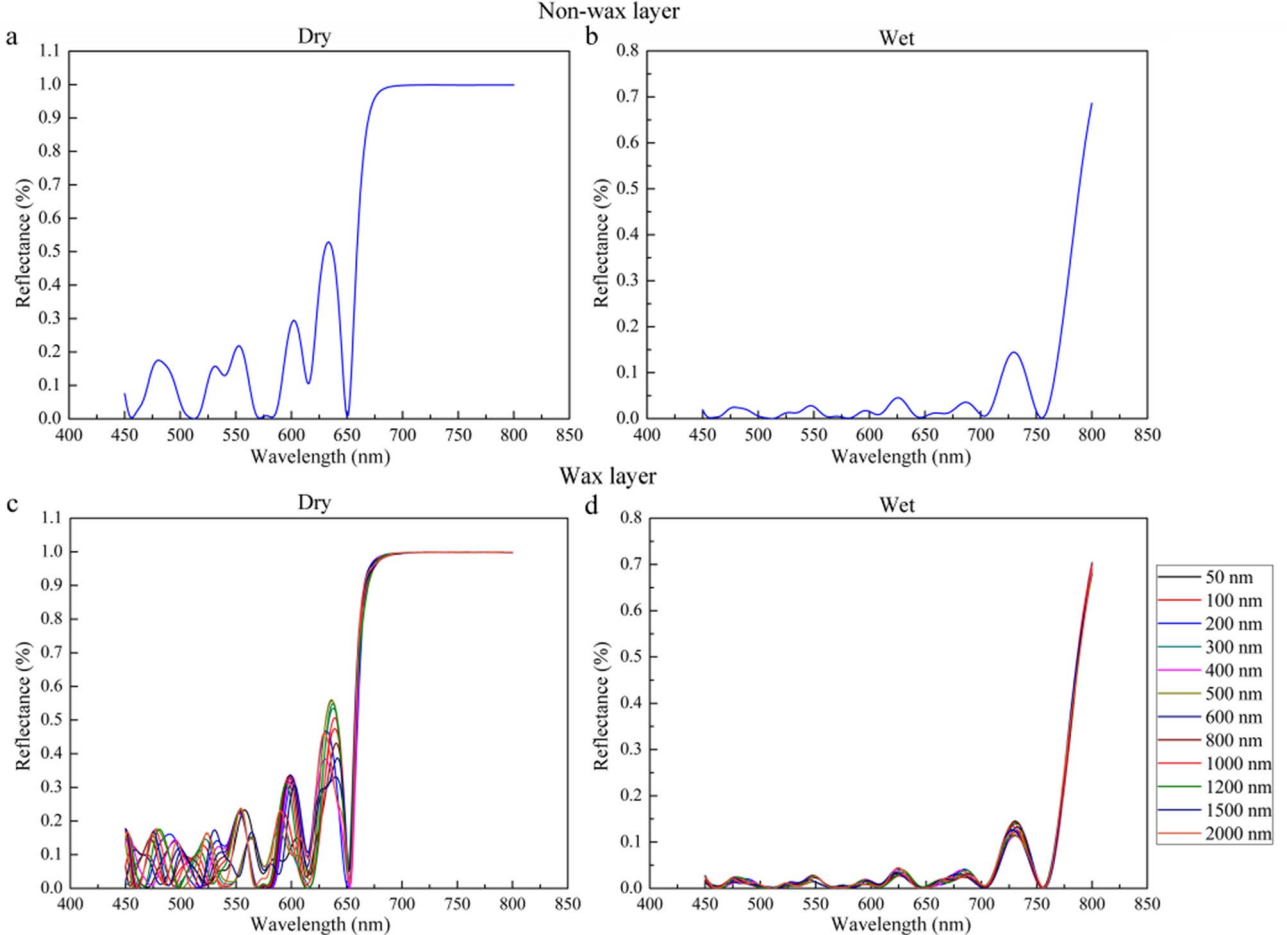
Simulated reflectance spectra of the multilayer unit model at an incidence angle of 0°. Non-wax layer in dry (**a**) and water-infiltrated (**b**) states. Addition of a wax layer on top of the outermost EPI and variation in its thickness in the dry (**c**) and water-infiltrated (**d**) states.

The reflectance spectra of the multilayer model were calculated by the transfer matrix method in a low-humidity state with no water and a high-humidity state filled with water, as shown in Fig. 5. For the purposes of the calculation, the high-index material was assumed to be the structural polymer chitin^26^. Then, for each unit, the multilayer structure consisted of two alternating layers of air and chitin, as shown in Fig. 1e. The air and chitin layer thicknesses in the spectrum calculation were obtained by dimensional measurements of the FIB-SEM images. The thicknesses of the EPI, air layer and chitin layer were 1479 ± 125 nm, 132 ± 14 nm and 157 ± 32 nm, respectively. The calculated reflectance results in the low-humidity state with no water, are presented in Fig. 5a. The refractive index used for the air layer was 1.00, and that of the chitin layer was 1.55^27–29^. To simulate the reflectance spectra of the multilayer structure filled with water in the high-humidity state, the air layer in the low-humidity state was replaced by a water layer with a refractive index of 1.33, as shown in Fig. 5b.

In the simulated results for a multilayer unit of elytron cuticle, the reflectance shows a fluctuating variation that is highly consistent with the testing results, and then the value curves of both states display a sudden increase. In addition to the similar fluctuation, the theoretically calculated reflectance values vary at wavelengths from 450 to 800 nm with no peaks. Meanwhile, the simulation results show that the reflectance of the yellow-green dry state is higher than that of the deep-brown state after water infiltration, as in the experimental results. Therefore, the cuticle microstructure can be shown to consist of numerous multilayer units. In addition, water infiltration will increase light absorption, resulting in lower reflectance that is the determining factor in the color change to deep-brown.

The presence of wax is not the only determinant of hydrophobic properties but rather acts in combination with microscopic structures found on the surface^30^. The wax layer on the cuticle surface has been reported to increase its reflectance^31,32^. To investigate the influence of the wax layer on reflectance, the reflectance spectra of the model multilayer unit was simulated with the addition of a wax layer, as shown in Fig. 5c,d. The refractive index of the wax surface layer was assumed to be 1.40, which is the value determined for the cuticular wax on dragonfly wings^33^. The simulated thickness of this layer was varied from 50 to 2000 nm. Figure 5c shows the calculated reflectance of the air-chitin multilayer unit in the dry state. No regular variation tendency or obvious differences in these simulated reflectance results can be observed, which implies that the thickness of the wax layer does not appear to influence the reflective spectrum, even at the maximum thickness of 2000 nm. Comparison with the results in Fig. 5a shows no significant difference between the reflectance with a waxy layer and that with no waxy layer. Figure 5d gives the calculated results for the water-chitin multilayer in a high-humidity state. As in the dry state, the reflectance curves overlay the original curve while the thickness of the wax layer varies from a minimum of 50 nm to a maximum of 2000 nm. Moreover, the waxy layer added on top of the EPI has no impact on the reflective spectra when compared to the simulated results without this layer as the air layer of the multilayer unit is filled with water through the wax.

## Discussion

### Dynamics of water absorption and wettability

When damp papers are placed on the elytron, water molecules begin to move through the EPI channels and then to fill the pores in the microstructure along the path of SUs under the covered area, whereas the water molecules do not enter the part that is not covered with wet paper. Thus, the SU in the EXO plays a role in achieving correct and distinct local changes in color. The insect cuticle is well known to be a layered composite consisting of chitin fibers and a proteinaceous matrix^34,35^. The SU is also part of the layered structure. Our previous paper^23^ examined the relationship between the mechanical properties of elytron materials and their reversible color changes. The elytra become less stiff and exhibit stronger viscous damping as the color becomes deeper. Combined with these changes in material properties, this specific mode of local color change may allow the beetle to disguise itself with various reversible color change patterns for flexible protection.

The left and right contact angles of a droplet as it first contacts the surface are 91.4° and 92.1°, respectively. Chitin is a rigid, permeable polymer that is the main constituent of beetle cuticle^36^. The static contact angle formed by a water droplet on a flat chitin surface is 102°, as calculated by Young’s equation^37^. However, the measured contact angle is smaller than the calculated result. In addition to chitin compounds, the waterproofing ability of beetle cuticle depends on the surface morphology. The many pores on the surface increase the roughness of the cuticle, generally increasing the wettability and decreasing the contact angle. The change in the contact angle is a phenomenon of wetting induced by intermolecular interactions when a water droplet contacts the elytron. When the attractive forces exerted by the solid molecules on the liquid molecules are greater than the attractive forces between the liquid molecules, the liquid surface shows a tendency toward diffusion, which results in the wetting phenomenon^38^. The degree of wetting is determined by the balance between adhesive and cohesive forces^39^. Moreover, liquid will permeate into the solid through capillary because of capillary effects during wetting^40^. As observed by Fig. 1e,f, there are many channels in the elytron cuticle connecting the exterior to the inner microstructure. These channels act as capillaries that allow the water on the elytron to penetrate through the envelope into the scale. Thus, the special microstructure and pores are the basis of the reversible color change of the beetle elytron, allowing water molecules to permeate in a high-humidity environment and evaporate in a low-humidity environment.

In addition to the pore morphology, the chemical composition is a factor in determining the water contact angle of the beetle elytron^41^. The elytron contains acids and salts with metallic elements. These salts and acids are known to dissolve well in water. The presence of the listed metallic and non-metallic elements could be critical in reducing the contact angle on the cuticle, since the water droplet will be more adhesive on account of the intermolecular forces between the salts and the water molecules. These interactions increase the attractive forces exerted by the solid molecules on the liquid molecules, resulting in a smaller contact angle than the calculated value. This special surface of the elytron may be related to the functions that protect the insect against water evaporation^42^, prevent too many water droplets from adhering and interfering with flight, and allow a little water permeating the elytron to change its color.

### Mechanism of reversible color-changing

The color changes of beetle elytra induced by soaking in water have previously been explained as the change in refractive index change upon filling of the air space in the microstructure with water^4,15^. *D. tityus* changes from yellow-green to deep-brown after water infiltration. The results of FIB and its three-dimensional reconstruction of the microstructure suggest that wetting the elytron cuticle will induce the replacement of air by water in the microstructure. The refraction angle of light in water is smaller than that in air. While the refractive index of air is 1.00, and that of water is 1.33, the refraction angle of light will decrease as the air space fills with water. The reflection of light will then be different, with a smaller refraction angle, resulting in greater light absorption and decreased reflectance. Furthermore, the reflectance increases gradually as the incidence angle increases from 0° to 60° at angular intervals of 5°. This change in reflectance may result from the microstructure of the beetle cuticle, which has a great effect on the path of light propagation. When the incident angle of light changes, the path by which light propagates in the microstructure is different. The greater the angle of incidence, the less light is absorbed after passing through the interior structure. Accordingly, greater reflectance is obtained. The reflectance of the deep-brown elytron soaked with water shows slower growth than that of the yellow-green elytron, which is associated with both the interior microstructure and the transformation of the refractive index. The change in the refractive index from 1.00 to 1.33 counteracts the effects of the incidence angle, which would tend to reduce the light absorption. Although the increase in the incident angle can reduce the absorption of light, some light will be still absorbed when refracted by water in the microstructure of the elytron during the spreading process. Therefore, the transformation of the refractive index and the microstructure are essential factors in the reversible color change of *D. tityus*.

Despite the agreements between the calculated and experimental spectra, it is noticeable that the calculated values of the fluctuating region are much lower than the experimental values. In the natural multilayer system, the refractive index and the thickness of individual layers determine the reflectance in a thin-film interference model^43^. However, even for the known constituents of chitin, pigments and protein, information about their real refractive index is generally lacking^44^. In the calculations of Maxwell’s equations in this study, the measured average thickness and a widely recognized value of the refractive index are used. The unavoidable variation in thickness will influence the reflectance of each wavelength, though the exact values are unknown. On the other hand, the calculated results are based on a regular multilayer structure that may differ slightly from the real microstructure. Thus, the absorption of light in the model and real microstructures will be different, which could cause the difference between the experimental and calculated results in the reflective spectrum. According to the simulation results, the waxy layer has no effect on the reflection of light. The data obtained in this investigation are radically different from those in previous studies. Wax may not need to affect light reflection to achieve a reversible color change. In that case, the role of the wax layer of *D. tityus* may simply be to influence the hydrophobic properties and protect the beetle from desiccation.

## Conclusion

Based on focused ion beam-scanning electron microscopy (FIB-SEM), the three-dimensional microstructure of the *D. tityus* elytron was revealed. The epicuticle (EPI) is a permeable layer, and the exocuticle (EXO) is a three-dimensional photonic crystal. Contact angle, surface chemical composition and optical experiments were used to investigate the characteristics and mechanism of the reversible color change. The contact angle is greater than 90° when a water droplet first falls onto the elytron surface. The presence of specific elements on the surface and channels in the EPI then cause the water droplet to become more adhesive and begin to permeate into the elytron. The special microstructure and surface chemical composition are the basis for the reversible color change of the beetle elytron. Moreover, structural units (SUs) exist in the EXO to allow accurate and distinct local color changes. The reflectance of both yellow-green and deep-brown elytra displays a gradual increase as the incidence angle increases from 0° to 60°. The microstructure and the transformation of the refractive index are the key factors in the process of reversible color change in *D. tityus*. The simulated results suggest that water infiltration increases light absorption, resulting in lower reflectance, which is the determining factor in the color change to deep-brown. The microstructure of the cuticle consists of numerous multilayer units. In contrast, the waxy layer has no effect on the reflection of light.
